# Optimal Sixteenth Order Convergent Method Based on Quasi-Hermite Interpolation for Computing Roots

**DOI:** 10.1155/2014/410410

**Published:** 2014-08-12

**Authors:** Fiza Zafar, Nawab Hussain, Zirwah Fatimah, Athar Kharal

**Affiliations:** ^1^Centre for Advanced Studies in Pure and Applied Mathematics (CASPAM), Bahauddin Zakariya University, Multan 60800, Pakistan; ^2^Department of Mathematics, King Abdulaziz University, P.O. Box 80203, Jeddah 21589, Saudi Arabia; ^3^National University of Sciences and Technology (NUST), Islamabad 44000, Pakistan

## Abstract

We have given a four-step, multipoint iterative method without memory for solving nonlinear equations. The method is constructed by using quasi-Hermite interpolation and has order of convergence sixteen. As this method requires four function evaluations and one derivative evaluation at each step, it is optimal in the sense of the Kung and Traub conjecture. The comparisons are given with some other newly developed sixteenth-order methods. Interval Newton's method is also used for finding the enough accurate initial approximations. Some figures show the enclosure of finitely many zeroes of nonlinear equations in an interval. Basins of attractions show the effectiveness of the method.

## 1. Introduction

Let us consider the problem of approximating the simple root x* of the nonlinear equation involving a nonlinear univariate function *f*:

(1) $$\begin{array}{c} f\left(x\right)=0. \end{array}$$

Newton's method and its variants have always remained as widely used one-point without memory and one-step methods for solving (1). However, the usage of single point and one-step methods puts limit on the order of convergence and computational efficiency is given as

(2) $$\begin{array}{c} E=p^{1/\theta }, \end{array}$$

where *p* is the order of convergence of the iterative method and *θ* is the cost of evaluating *f* and its derivatives.

To overcome the drawbacks of one-point, one-step methods, many multipoint multistep higher order convergent methods have been introduced in the recent past by using inverse, Hermite, and rational interpolation [1, 2]. In developing these methods, so far, the conjecture of Kung and Traub has remained the focus of attention. It states the following.

**Conjecture 1 .**

An optimal iterative method without memory based on n evaluations would achieve an optimal convergence order of 2^*n*−1^, hence, a computational efficiency of 2^(*n*−1)/*n*^.

In [3, 4], Petkovi$\overset{´}{\text{c}}$ presented a general optimal *n*-point iterative scheme without memory defined by

(3) xk+1=ϕn(xk)=Nn−1(Nn−2(⋯(N2(ψf(xk)))⋯)),k=0,1,2,…,

where *x*_*k*_ is the approximation of the root x* at the *k*th iteration and *ψ*_*f*_(*x*_*k*_) = *ϕ*_2_(*x*_*k*_) is an arbitrary fourth-order, two-point method requiring three function evaluations:

(4) $$\begin{array}{c} \phi_{m+1}\left(x_{k}\right)=N_{m}\left(\phi_{m}\right)=\phi_{m}-\frac{f\left(\phi_{m}\right)}{f^{\prime }\left(\phi_{m}\right)},\;m=2,\ldots ,n-1 \end{array}$$

is Newton's method. The derivative at *m* + 1-step is approximated through quasi-Hermite interpolatory polynomial of degree *m* + 1, denoted by *h*_*m*+1_′(*ϕ*_*m*_).

Using this approach, Sargolzaei and Soleymani [5] presented a three-step optimal eighth-order iterative method. However, since the authors approximated the derivative at the fourth step by using Hermite interpolatory polynomials of degree three, therefore the fourth-step method given by Sargolzaei and Soleymani has order of convergence fourteen including five function evaluations, which is not optimal in the sense of Kung and Traub.

In this paper, we present an optimal four-step four-point sixteenth-order convergent method by using quasi-Hermite interpolation from the general class of Petkovi$\overset{´}{\text{c}}$ [3, 4]. The interpolation is done by using the Newtonian formulation given by Traub [6]. The numerical comparisons are given in Section 4 with recent optimal sixteenth-order convergent methods based on rational interpolants. Since, the first step of our method is Newton's method, thus to overcome the drawbacks of Newton's method we have calculated, in Section 5, accurate initial guess required for the convergence of this method for some oscillatory functions.

## 2. Construction of Method

We define the following:

(5) $$\begin{array}{c} y_{n}=x_{n}-\frac{f\left(x_{n}\right)}{f^{\prime }\left(x_{n}\right)},\\ z_{n}=\psi_{f}\left(x_{n},y_{n}\right),\\ t_{n}=\varphi_{f}\left(x_{n},y_{n},z_{n}\right),\\ x_{n+1}=t_{n}-\frac{f\left(t_{n}\right)}{f^{\prime }\left(t_{n}\right)}, \end{array}$$

where *ψ*_*f*_(*x*_*n*_, *y*_*n*_) and *φ*_*f*_(*x*_*n*_, *y*_*n*_, *z*_*n*_) are any arbitrary fourth- and eighth-order, multipoint methods. We, now, approximate *f*′(*t*_*n*_) with a quasi-Hermite interpolatory polynomial of degree four satisfying

(6) $$\begin{array}{c} f\left(x_{n}\right)=h_{4}\left(x_{n}\right),\\ f^{\prime }\left(x_{n}\right)=h_{4}^{\prime }\left(x_{n}\right),\\ f\left(y_{n}\right)=h_{4}\left(y_{n}\right),\\ f\left(z_{n}\right)=h_{4}\left(z_{n}\right),\\ f\left(t_{n}\right)=h_{4}\left(t_{n}\right). \end{array}$$

To construct the interpolatory polynomial *h*_4_(*t*), satisfying the above conditions, we apply the Newtonian representation of the interpolatory polynomial satisfying the conditions

(7) P(kj)(xi−j)=f(kj)(xi−j), kj=0,1,…,γj−1,γj≥1, j=0,1,2,3,….

Traub [6, p. 243] have given this as follows:

(8) h4(t)≡P3,γ(t)=∑j=03∑l=0γj−1Cl,jγj(t)f[xi,γ0;xi−1,γ;…;xi−j,l+1],

(9) $$\begin{array}{c} C_{l,j}^{\gamma_{j}}\left(t\right)={\left(t-x_{i-j}\right)}^{l}\overset{j-1}{\underset{k=0}{\prod }}{\left(t-x_{i-k}\right)}^{\gamma_{k}},\;\;\overset{-1}{\underset{0}{\prod }}=1. \end{array}$$

The confluent divided differences involved here are defined as

(10) f[xi,γ0;…;xi−q,γq;…;xi−r,γr;…;xi−n,γn]=1(xi−q−xi−r) ×(f[xi,γ0;…;xi−q,γq;…;xi−r,γr−1;…;xi−n,γn]−f[xi,γ0;…;xi−q,γq−1;…;xi−r,γr;…;xi−n,γn]),f[xi−j;l+1]=f(l)(xi−j)l!.

In particular, *f*[*x*_*i*−*j*_, 1] = *f*[*x*_*i*−*j*_] is the usual divided difference. Here, we take *x*_*i*_ = *t*_*n*_, *x*_*i*−1_ = *z*_*n*_, *x*_*i*−2_ = *y*_*n*_, *x*_*i*−3_ = *x*_*n*_ and hence, *γ*_0_ = 1, *γ*_1_ = 1, *γ*_2_ = 1, and *γ*_3_ = 2. Expanding (8), we get

(11) h4(t)=f(tn)+(t−tn)f[tn,zn] +(t−tn)(t−zn)f[tn,zn,yn] +(t−tn)(t−zn)(t−yn)f[tn,zn,yn,xn] +(t−tn)(t−zn)(t−yn)(t−xn)f[tn,zn,yn,xn,2].

Differentiating (11) with respect to “*t*” and substituting *t* = *t*_*n*_ in the above equation, we obtain

(12) h4′(tn)=f[tn,zn]+(tn−zn)f[tn,zn,yn] +(tn−zn)(tn−yn)f[tn,zn,yn,xn] +(tn−zn)(tn−yn)(tn−xn)f[tn,zn,yn,xn,2],

where

(13) f[tn,zn,yn]=1(tn−yn)(f[tn,zn]−f[tn,yn]),f[tn,zn,yn,xn] =1(tn−xn)(tn−yn)(f[tn,zn]−f[zn,yn])  −1(tn−xn)(zn−xn)(f[zn,yn]−f[yn,xn]),f[tn,zn,yn,xn,2] =1(tn−xn)2(tn−yn)(f[tn,zn]−f[zn,yn])  −1(tn−xn)2(zn−xn)(f[zn,yn]−f[yn,xn])  −1(zn−xn)2(tn−xn)(f[zn,yn]−f[yn,xn])  +1(tn−xn)(zn−xn)(yn−xn)  ×(f[yn,xn]−f′(xn)).

Using representation (12) of *h*_4_′(*t*_*n*_) in place of *f*′(*t*_*n*_) at the fourth step, the new four-step iterative method is obtained as

(14) $$\begin{array}{c} z_{n}=\psi_{f}\left(x_{n},y_{n}\right),\\ t_{n}=\varphi_{f}\left(x_{n},y_{n},z_{n}\right),\\ x_{n+1}=t_{n}-\frac{f\left(t_{n}\right)}{h_{4}^{\prime }\left(t_{n}\right)}, \end{array}$$

where *ψ*_*f*_(*x*_*n*_, *y*_*n*_) and *φ*_*f*_(*x*_*n*_, *y*_*n*_, *z*_*n*_) are any fourth- and eighth-order convergent methods, respectively, and

(15) h4′(tn)=f[tn,zn]+(tn−zn)f[tn,zn,yn] +(tn−zn)(tn−yn)f[tn,zn,yn,xn] +(tn−zn)(tn−yn)(tn−xn)f[tn,zn,yn,xn,2].

**Theorem 2 .**

Let one consider x* as a root of nonlinear equation (1) in the domain *D* and assume that *f*(*x*) is sufficiently differentiable in the neighbourhood of the root. Then the iterative method defined by (14) is of optimal order sixteen and has the following error equation:

(16) $$\begin{array}{c} e_{n+1}=x_{n+1}-x^{*}=-c_{2}b_{1}\left(a_{1}c_{5}-b_{1}\right)e_{n}^{16}+O\left(e_{n}^{17}\right), \end{array}$$

where *c*_*i*_, for *i* ≥ 2, are defined by

(17) $$\begin{array}{c} c_{i}=\frac{1}{i!}\left(\frac{f^{\left(i\right)}\left(x^{*}\right)}{f^{\prime }\left(x^{*}\right)}\right),\;i=0,1,2,3,\ldots . \end{array}$$

**Proof**

We write the Taylor series expansion of the function *f* about the simple root x* in *n*th iteration. Let *e*_*n*_ = *x*_*n*_ − x*. Therefore, we have

(18) f(xn)=f′(x∗)[en+c2en2+c3en3+c4en4+c5en5+  c6en6+c7en7+c8en8+O(en9)].

Also, we obtain

(19) f′(xn)=f′(x∗)[1+2c2en+3c3en2+4c4en3+5c5en4+6c6en5+7c7en6+8c8en7+O(en8)].

Now, we find the Taylor expansion of *y*_*n*_, the first step, by using the above two expressions (18) and (19). Hence, we have

(20) yn=c2en2+(2c3−2c22)en3+(3c4−7c2c3+4c23)en4 +(4c5−10c2c4−6c32+20c3c22−8c24)en5+O(en6).

Also, we need the Taylor expansion of *f*(*y*_*n*_); that is

(21) f(yn)=f′(x∗) ×[c2en2−2(c22−c3)en3+(3c4−7c2c3+5c23)en4−2(−2c5+5c2c4+3c33−12c3c22+6c24)en5+O(en6)].

In second step, we take a general fourth-order convergent method as

(22) zn=a1en4+a2en5+a3en6+a4en7+a5en8+O(en9),  f(zn)=f′(x∗)[a1en4+a2en5+a3en6 +  a4en7+(c2a12+a5)en8+O(en9)].

Now, we find the Taylor expansion of each divided difference used at the third step. We thus obtain

(23) f[xn,yn]  =f′(x∗)[1+c2en+(c3+c22)en2+(c4+3c2c3−2c23)en3+⋯+O(en9)],f[xn,zn]=f′(x∗)[1+c2en+c3en2+c4en3+(c5+c2a1)en4+⋯+O(en9)],f[yn,zn]=f′(x∗)[1+c22en2−2c2(−c3+c22)en3+c2(3c4−6c2c3+4c23+a1)en4+⋯+O(en9)],f[yn,xn,xn]=f′(x∗)[c2+2c3en+(3c4+c2c3)2en2+(2c2c4−2c3c22+4c5+2c32)en3+⋯+O(en9)].

In the third step, we take a general eighth-order convergent method as follows:

(24) $$\begin{array}{c} t_{n}=b_{1}e_{n}^{8}+b_{2}e_{n}^{9}+b_{3}e_{n}^{10}+b_{4}e_{n}^{11}+b_{5}e_{n}^{12}+\cdots +O\left(e_{n}^{17}\right), \end{array}$$

and the Taylor expansion for *f*(*t*_*n*_) is

(25) f(tn) =f′(x∗)  ×[b1en8+b2en9+b3en10+b4en11+b5en12+⋯+O(en17)].

Now, we find the Taylor expansion of divided differences used at the last step. We, thus, obtain

(26) f[tn,zn,yn]=f′(x∗)[c2+c2c3en2−2c3(−c3+c22)en3+⋯+O(en17)],f[tn,zn,yn,xn]=f′(x∗)[c3+c4en+(c2c4+c5)en2+(2c3c4−c22c4+c6+c2c5)en3+⋯+O(en17)],f[tn,zn,yn,xn,2]=f′(x∗)[c4+2c5en+(3c6+c2c5)en2+(2c2c6+4c7+2c3c5−2c22c5)en3+⋯+O(en17)].

Hence, our fourth step defined in (14) becomes

(27) $$\begin{array}{c} x_{n+1}=-c_{2}b_{1}\left(a_{1}c_{5}-b_{1}\right)e_{n}^{16}+O\left(e_{n}^{17}\right), \end{array}$$

which manifests that (14) is a four-step iterative method of optimal order of convergence of sixteen consuming four function evaluations and one derivative evaluation.

**Remark 3 .**

It is concluded from Theorem 2 that the new sixteenth-order convergent iterative method (14) for solving nonlinear equations satisfies the conjecture of Kung and Traub that a multipoint method without memory with four evaluations of functions and a derivative evaluation can achieve an optimal sixteenth order of convergence (2^4^ = 16) and an efficiency index of 2^4/5^ = 1.741.

## 3. Some Particular Methods

In this section, we consider some particular methods from the newly developed family of the sixteenth-order convergent iterative methods.

### 3.1. Iterative Method M1

Here, we take *ψ*_*f*_(*x*_*n*_, *y*_*n*_) as two-step fourth-order convergent method defined by Geum and Kim [7] and the third-step *φ*_*f*_(*x*_*n*_, *y*_*n*_, *z*_*n*_) is replaced by the third step of eighth-order convergent method given by [5] using Hermite interpolation. Hence, our four-step method becomes

(28) yn=xn−f(xn)f′(xn),zn=yn−(1+f(yn)f(xn))2f(yn)f′(xn),tn=zn−f(zn) ×(2f[xn,zn]+f[yn,zn]−2f[xn,yn]  +(yn−zn)f[yn,xn,xn])−1,      xn+1=tn−f(tn)h4′(tn),

where *h*_4_′(*t*_*n*_) is given by (15).

### 3.2. Iterative Method M2

Here, we define *ψ*_*f*_(*x*_*n*_, *y*_*n*_) as King's two-step fourth-order convergent method [8] with *β* = 0, as

(29) $$\begin{array}{c} y_{n}=x_{n}-\frac{f\left(x_{n}\right)}{f^{\prime }\left(x_{n}\right)},\\ z_{n}=y_{n}-\frac{f\left(y_{n}\right)}{f^{\prime }\left(x_{n}\right)}\frac{f\left(x_{n}\right)}{f\left(x_{n}\right)-2f\left(y_{n}\right)}. \end{array}$$

Hence, our four-step iterative method becomes

(30) yn=xn−f(xn)f′(xn),zn=yn−f(yn)f′(xn)f(xn)f(xn)−2f(yn),tn=zn−f(zn) ×(2f[xn,zn]+f[yn,zn]−2f[xn,yn]  +(yn−zn)f[yn,xn,xn])−1,          xn+1=tn−f(tn)h4′(tn),

where *h*_4_′(*t*_*n*_) is given by (15).

## 4. Numerical Results and Computational Cost

In this section, we compare our newly constructed family of iterative methods of optimal sixteenth-order M1 and M2 defined in (28) and (30), respectively, with some famous equation solvers. For the sake of comparison, we consider the fourteenth-order convergent method (PF) given by Sargolzaei and Soleymani [5] and the optimal sixteenth-order convergent methods (JRP) and (FSH) given by Sharma et al. [1] and Soleymani et al. [2], respectively. All the computations are done using software Maple 13 with tolerance *ε* = 10^−1000^ and 4000 digits precision. The stopping criterion is

(31) $$\begin{array}{c} \left|f\left(x_{n}\right)\right|<\varepsilon . \end{array}$$

Here, x* is the exact zero of the function and *x*_0_ is the initial guess. In Tables 1–9, columns show the number of iterations *n*, in which the method converges to x*, the absolute value of function |*f*(*x*_*n*_)| at *n*th step, for *n* = 1,2, 3. The numerical examples are taken from [1, 2].

We now give the numerical results of our new schemes in comparison with Newton's method for three oscillatory nonlinear functions, *f*_7_(*x*) = −cos⁡(2 − *x* ^2^) + log⁡(*x*/7)+(1/10) in the domain [1,15] having 69 zeroes, *f*_8_(*x*) = (*x* ^2^ − 4)sin  (100*x*) on the interval [0,10] having 320 zeroes, and *f*_9_(*x*) = *e* ^sin  (log⁡(*x*)cos⁡  (20*x*))^ − 2 in the domain [2,10] having 51 zeroes using the same precision, stopping criterion, and tolerance as given above. The first two functions *f*_7_(*x*) and *f*_8_(*x*) are taken from [9] and *f*_9_(*x*) is taken from [2].  Table 8 shows the importance of accurate initial guesses for the convergence of Newton's method (NM) for these types of highly fluctuating functions. The results include the number of iterations *n*, the absolute value of each function at the *n*th iterate |*f*(*x*_*n*_)|, and the root x* to which the methods converge.

Table 9 shows the cost of executing each method for solving a nonlinear equation. The table clearly depicts that except that of the fourteenth-order convergent method given by Sargolzaei and Soleymani (PF) [5] all other methods of respective domain require more computational effort compared to our methods M1 and M2.

## 5. Newton's Method and Zeroes of Functions

The new sixteenth-order iterative method developed in this paper includes Newton's method as the first step. Although Newton's method is one of the most widely used methods, still it has many drawbacks; that is, proper initial guess plays a crucial role in the convergence of this method; an initial guess, which is not close enough to the root of the function, may lead to divergence as shown in Table 8. Moreover, another drawback is the involvement of derivative which may not exist at some points of the domain. To overcome these two main drawbacks of Newton's method, Moore et al. in 1966 ([10], Chapter 9) gave a method called interval Newton's method which can generate the safe initial guesses to ensure the convergence of Newton's method in vicinity of the root. However, interval Newton's method for handling nonlinear equations has a restriction that if the interval extension of initial guess *X* ^(0)^ contains a zero of the function *f*′(*x*), then every *k*th iteration *X* ^(*k*)^ contains the zero of *f*′(*x*) for all *k* = 0,1, 2,3,…, which thus leads to failure of this method. Thus, *X* ^(*k*)^ forms a nested sequence converging to *x* only if 0 ∉ *F*′(*X* ^(0)^). To remove this restriction and to allow the range of values of the derivative *f*′(*x*) to contain zero, Moore et al. ([10], Chapter 5) gave an extension of this method by splitting the quotient *f*(*x*)/*f*′(*X*) occurring in interval Newton's method into two subintervals, where each subinterval though contains a zero of the function but excludes the zero of the derivative of *f*(*x*). This method is known as extended interval Newton's method. We, herein, find the intervals enclosing all the zeroes of the function by using extended interval Newton's method defined in [10]. The endpoints of these subintervals are approximated up to 10 decimal places which may serve as initial guesses, good enough to show convergence for all the zeroes of oscillatory nonlinear functions.

By using Maple, we find the subintervals for *f*_7_(*x*), *f*_8_(*x*), and *f*_9_(*x*) defined above in Section 4. For *f*_7_(*x*), 69 subintervals are calculated as follows:

(32) [14.91409469,14.91907799],[14.87618033,14.87882406],[14.70253913,14.70832763],[14.66422750,14.66485977],[14.48793933,14.49176427],[14.44786835,14.44826560],[14.26995241,14.27656721],[14.22604037,14.23066461],[14.04552680,14.05497489],[14.00282091,14.00616288],[13.81848674,13.82996307],[13.77545098,13.77899594],[13.59080017,13.60161684],[13.54513440,13.54762734],[13.36096809,13.36958249],[13.31111271,13.31195699],[13.12910597,13.13311931],[13.06857536,13.07318658],[12.88867541,12.89225623],[12.82502646,12.82959150],[12.64414769,12.64629550],[12.57794309,12.58168834],[12.39221606,12.39604347],[12.32427994,12.33155620],[12.13933578,12.14152125],[12.06589114,12.06949432],[11.87681567,11.88836316],[11.80257913,11.80785022],[11.61017387,11.61417336],[11.52580093,11.53582338],[11.33855318,11.34565854],[11.25371239,11.25707075],[11.05782638,11.06375050],[10.97106243,10.97617210],[10.77402365,10.77570682],[10.67229899,10.68264897],[10.48006360,10.48312670],[10.37979979,10.38123836],[10.17800639,10.17943511],[10.07090553,10.07387957],[9.866768415,9.868206263],[9.752713256,9.754776613],[9.542656144,9.546467439],[9.423111972,9.425145592],[9.209671918,9.212909453],[9.081311827,9.085205858],[8.866105341,8.868806136],[8.723516543,8.727512993],[8.507376755,8.509833917],[8.355789454,8.356573732],[8.133209509,8.133391926],[7.967541337,7.968419812],[7.740477001,7.741246284],[7.560115543,7.560177140],[7.327235639,7.327826668],[7.128324205,7.128337654],[6.889745558,6.889772255],[6.667942072,6.668081225],[6.422938778,6.423097661],[6.172706697,6.173230855],[5.917397368,5.920842381],[5.632674465,5.632904614],[5.372683988,5.375017239],[5.031577748,5.033245710],[4.765041785,4.765502794],[4.346558913,4.346610460],[4.073130785,4.073647556],[3.516479084,3.516796449],[3.253019201,3.253316980].

Likewise, for the nonlinear function *f*_8_(*x*), the interval [0,10] is subdivided into 320 subintervals given as

(33) [9.990184044,10],[9.955946724,9.959695063],[9.921726460,9.927528356],[9.895991269,9.901813845],[9.860932807,9.867395738],[9.833013483,9.842299773],[9.798356795,9.802371500],[9.764625542,9.770416585],[9.738902076,9.744714230],[9.703232575,9.709925323],[9.675902793,9.685025935],[9.641179727,9.645245491],[9.607542832,9.613332772],[9.581821638,9.587633073],[9.546128339,9.552831077],[9.518599577,9.527156111],[9.483395827,9.487943109],[9.450441211,9.456225830],[9.424726523,9.430533040],[9.388258511,9.395312094],[9.361797477,9.362112523],[9.327254150,9.331188503],[9.293391113,9.299187867],[9.267665764,9.273484771],[9.232219640,9.238827421],[9.204564556,9.213401510],[9.169580781,9.173885974],[9.136290459,9.142079783],[9.110573681,9.116385739],[9.074453010,9.081345324],[9.047440909,9.056141780],[9.012398012,9.016770408],[8.979207819,8.984996872],[8.953492707,8.959304932],[8.917330060,8.924242464],[8.890031492,8.898238370],[8.854549244,8.859511211],[8.822106558,8.827894687],[8.796393961,8.802205670],[8.759426649,8.766743202],[8.733620068,8.737367181],[8.700194918,8.703778580],[8.665140340,8.670997704],[8.639361807,8.645242888],[8.605076793,8.611338461],[8.576524492,8.576819799],[8.542723242,8.546366963],[8.508031342,8.513867105],[8.482275207,8.488135298],[8.447441739,8.453855727],[8.419442565,8.419733454],[8.385642791,8.389273297],[8.350952363,8.356789480],[8.325196573,8.331058521],[8.290472928,8.296852876],[8.262306529,8.262599657],[8.227975566,8.231819589],[8.193839383,8.199655788],[8.168105345,8.173947253],[8.132631531,8.139267983],[8.105301657,8.108754352],[8.071887350,8.075485577],[8.036821970,8.042687844],[8.011041930,8.016933569],[7.976738061,7.983014238],[7.948206312,7.948506710],[7.914414622,7.918069963],[7.879712050,7.885556070],[7.853954900,7.859825409],[7.819100301,7.825530412],[7.791127469,7.791426462],[7.757383380,7.761016594],[7.722636327,7.728484673],[7.696876988,7.702752410],[7.662184799,7.668566032],[7.633999479,7.634293225],[7.599741530,7.603562482],[7.565522523,7.571349577],[7.539785779,7.545640712],[7.504367753,7.510993040],[7.473598085,7.477903415],[7.445535699,7.445741224],[7.409526765,7.415709324],[7.376633149,7.382748116],[7.350628015,7.356769948],[7.316205621,7.320727946],[7.288433978,7.288634901],[7.252187667,7.258500302],[7.219382270,7.225665666],[7.193227785,7.199537852],[7.159100616,7.163628382],[7.131354615,7.131554070],[7.095214411,7.101441457],[7.062406422,7.068587545],[7.036345992,7.042555094],[7.001343483,7.006374052],[6.974208505,6.974430846],[6.937739087,6.944121686],[6.911502119,6.917912067],[6.878629934,6.885058820],[6.845557212,6.849712217],[6.817229629,6.817372349],[6.781328958,6.787520738],[6.748326672,6.754431355],[6.722341155,6.728475853],[6.688217195,6.692543011],[6.660135374,6.660349224],[6.624020054,6.630322401],[6.591095561,6.597348936],[6.564978649,6.571261929],[6.531350276,6.535539635],[6.503067732,6.503242835],[6.467150640,6.473357071],[6.434150065,6.440272246],[6.408154615,6.414308468],[6.373988633,6.378364826],[6.345971330,6.346185408],[6.314591592,6.314801811],[6.283130684,6.283189482],[6.251439225,6.251792397],[6.219732001,6.223771957],[6.188797959,6.189152266],[6.157480535,6.157523164],[6.120271452,6.126143816],[6.094516232,6.100424165],[6.062632401,6.066666282],[6.031713707,6.032062120],[6.000404602,6.000443419],[5.963159907,5.969063625],[5.937378306,5.937652790],[5.905762388,5.910128802],[5.874676316,5.875173796],[5.843267432,5.843366441],[5.811903897,5.811997045],[5.780439491,5.780578109],[5.748846678,5.753534709],[5.717642569,5.718446131],[5.686026171,5.686297214],[5.649589507,5.654965745],[5.623410417,5.623560314],[5.591995021,5.592085462],[5.560617933,5.560647031],[5.529050201,5.529379409],[5.494655569,5.498548325],[5.466333793,5.466594000],[5.434917226,5.435007765],[5.403538504,5.403560842],[5.371986243,5.372310224],[5.337627395,5.341510491],[5.309259066,5.309710904],[5.277842856,5.277931374],[5.246459480,5.246465096],[5.214949689,5.215260016],[5.180745879,5.184567812],[5.152175433,5.152483750],[5.120757973,5.120850212],[5.089379473,5.089393976],[5.057853016,5.058169633],[5.023599984,5.027444216],[4.995089639,4.995134322],[4.963669800,4.963761292],[4.931962692,4.932342959],[4.898577516,4.902594687],[4.869459724,4.871816598],[4.838049762,4.838119448],[4.806585813,4.806656799],[4.774807982,4.775256312],[4.740143422,4.744730700],[4.712320646,4.712554905],[4.680971224,4.681013199],[4.649505766,4.649581859],[4.617729602,4.618176851],[4.586717450,4.586839669],[4.555222666,4.555463069],[4.523886327,4.524034761],[4.492422625,4.492489967],[4.460687549,4.461092742],[4.429626928,4.429896951],[4.398061805,4.398325942],[4.366778548,4.366815736],[4.335349171,4.335447559],[4.303567341,4.304025136],[4.269958287,4.274126308],[4.241130124,4.241188163],[4.209730675,4.209803528],[4.178264652,4.178341647],[4.146497230,4.146938490],[4.115477159,4.115614121],[4.083976127,4.084225147],[4.052653043,4.052680415],[4.021185210,4.021271957],[3.989407736,3.989860744],[3.958398563,3.958520073],[3.926898618,3.927159520],[3.895568291,3.895695440],[3.864101345,3.864176810],[3.832368110,3.832776502],[3.801307500,3.801582245],[3.769733594,3.770018057],[3.737666515,3.738555787],[3.707036303,3.707258802],[3.675589856,3.675724815],[3.644022999,3.644272546],[3.612664809,3.613201841],[3.581242406,3.581427897],[3.549951324,3.550099814],[3.518316545,3.518639229],[3.487139359,3.487171164],[3.455421928,3.455797823],[3.424086427,3.424354779],[3.392871166,3.393040380],[3.361225117,3.361561856],[3.330058543,3.330091857],[3.298317177,3.298716019],[3.267222476,3.267259278],[3.235785801,3.235916398],[3.203947909,3.204476592],[3.173007152,3.173020607],[3.141578336,3.143519185],[3.109138236,3.110261707],[3.078714790,3.079011324],[3.047231464,3.047417939],[3.015713510,3.015956944],[2.984302733,2.984850517],[2.952829764,2.953121570],[2.921627101,2.921839766],[2.889886911,2.890332100],[2.858821551,2.858853753],[2.827044564,2.827488073],[2.795383138,2.796077943],[2.764547903,2.764847380],[2.732879774,2.733264371],[2.701691670,2.701782470],[2.670004074,2.670487492],[2.638703303,2.638964768],[2.607457669,2.607717839],[2.575554284,2.576183720],[2.544682213,2.544691854],[2.513257101,2.513304406],[2.481645703,2.482108351],[2.450259771,2.450475846],[2.418980937,2.419491975],[2.387353600,2.387757870],[2.356161477,2.356486619],[2.324746316,2.325055200],[2.293356484,2.293386906],[2.261862260,2.262729261],[2.229815864,2.230705785],[2.198955277,2.200863128],[2.167010175,2.167980140],[2.136137801,2.136331276],[2.104659623,2.106463624],[2.073438362,2.073482179],[2.041868337,2.042183784],[2.009607591,2.011490024],[1.999321992,2.000867238],[1.979075768,1.979403681],[1.947483742,1.948823227],[1.915715167,1.916831748],[1.884748724,1.885972930],[1.853395782,1.854072910],[1.821703180,1.822445009],[1.790679838,1.790831120],[1.759031149,1.759348389],[1.727645494,1.727906376],[1.696406397,1.696726589],[1.664770896,1.665247435],[1.633595310,1.633854746],[1.602189721,1.602326032],[1.570689948,1.570808034],[1.539356239,1.539522903],[1.507823837,1.508168009],[1.476527951,1.476727588],[1.445011590,1.445147118],[1.413454259,1.413736717],[1.382250070,1.382713354],[1.350528569,1.350962287],[1.319436348,1.319792183],[1.288006269,1.288122893],[1.256626812,1.256637997],[1.225208939,1.225321263],[1.193725250,1.193981936],[1.162375566,1.162556404],[1.130894263,1.130987988],[1.099210832,1.099581028],[1.068130784,1.068242783],[1.036648438,1.036884688],[1.005295008,1.005511280],[0.9738188397,0.9739004934],[0.9421678445,0.9424969036],[0.9110567268,0.9111176941],[0.8796104016,0.8797644727],[0.8482209514,0.8483759597],[0.8167520110,0.8168256101],[0.7850137932,0.7854241593],[0.7539774035,0.7540419814],[0.7225301690,0.7227499657],[0.6911404930,0.6913237876],[0.6596741127,0.6597407949],[0.6279686776,0.6283402978],[0.5969009283,0.5969287896],[0.5654760243,0.5654943094],[0.5340653229,0.5341890824],[0.5026034884,0.5026633333],[0.4708183984,0.4712683612],[0.4398218509,0.4398447467],[0.4084003236,0.4084110725],[0.3769864778,0.3771074902],[0.3455273844,0.3455812080],[0.3141592392,0.3141593917],[0.2827428736,0.2827564026],[0.2513252985,0.2513279205],[0.2199082241,0.2200133160],[0.1884516594,0.1884996544],[0.1570796264,0.1570796692],[0.1256635747,0.1256707687],[0.09424771546,0.09424778634],[0.06283185165,0.06283185330],[0.03141592644,0.03141592674],[0.,0.00003862611601].

Similarly, we find out that the function *f*_9_(*x*) has 51 zeroes in subintervals

(34) [9.989560628,9.992137465],[9.800306886,9.800592868],[9.676233039,9.681699494],[9.479678599,9.487508694],[9.361916677,9.366649388],[9.167915476,9.172598808],[9.047316788,9.053357092],[8.855747880,8.857383666],[8.732241020,8.737239224],[8.539956314,8.543464735],[8.421410405,8.426221913],[8.224527618,8.230816552],[8.106288875,8.111000572],[7.909883981,7.914797928],[7.790388775,7.796772894],[7.596487817,7.599819788],[7.478101387,7.482505409],[7.280935917,7.286251169],[7.165355153,7.170463555],[6.966512553,6.972261500],[6.851637227,6.855738225],[6.651964539,6.657088997],[6.536662954,6.542243912],[6.340108479,6.340747429],[6.223746207,6.227585293],[6.024753773,6.026603725],[5.910192772,5.914790060],[5.708719363,5.714342230],[5.596962948,5.601063643],[5.394445916,5.397139080],[5.285123399,5.287582209],[5.078257731,5.082630331],[4.970931153,4.974798339],[4.765173416,4.765494072],[4.658574438,4.662674653],[4.449250236,4.451201750],[4.346033601,4.348540346],[4.133210243,4.136240815],[4.033004562,4.035802815],[3.817884685,3.818161353],[3.722030098,3.724036626],[3.501280126,3.501552691],[3.409742652,3.412729435],[3.182864306,3.185008382],[3.100066834,3.100836733],[2.864997815,2.865471627],[2.789473642,2.791666167],[2.543047934,2.545369212],[2.484628981,2.484810607],[2.212216544,2.212720982],[2.188542314,2.188577325].

**Figure 1:**
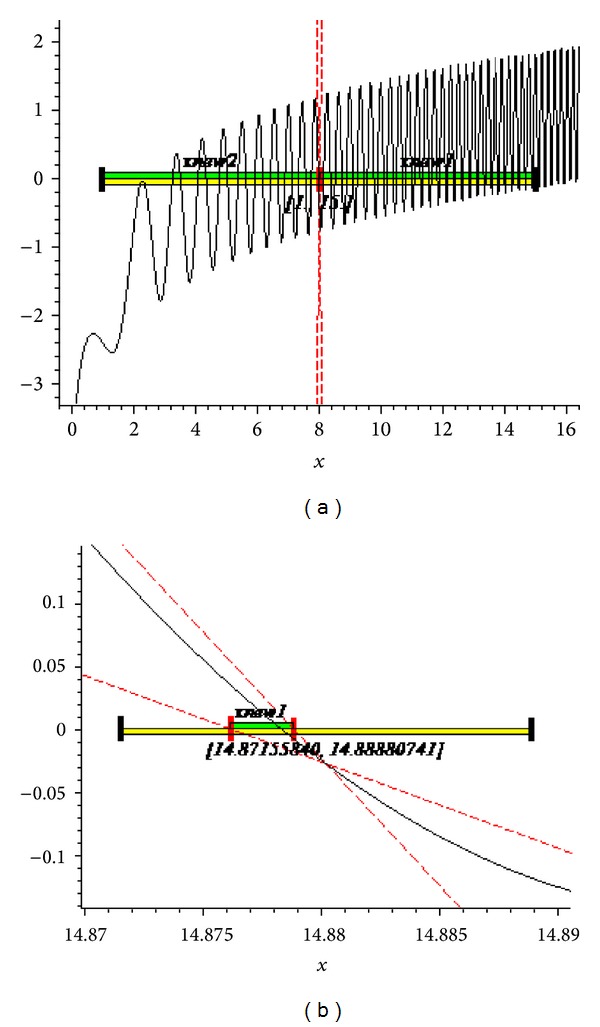
Graph of first and tenth iteration of extended interval Newton's method for *f*_7_(*x*).

**Figure 2:**
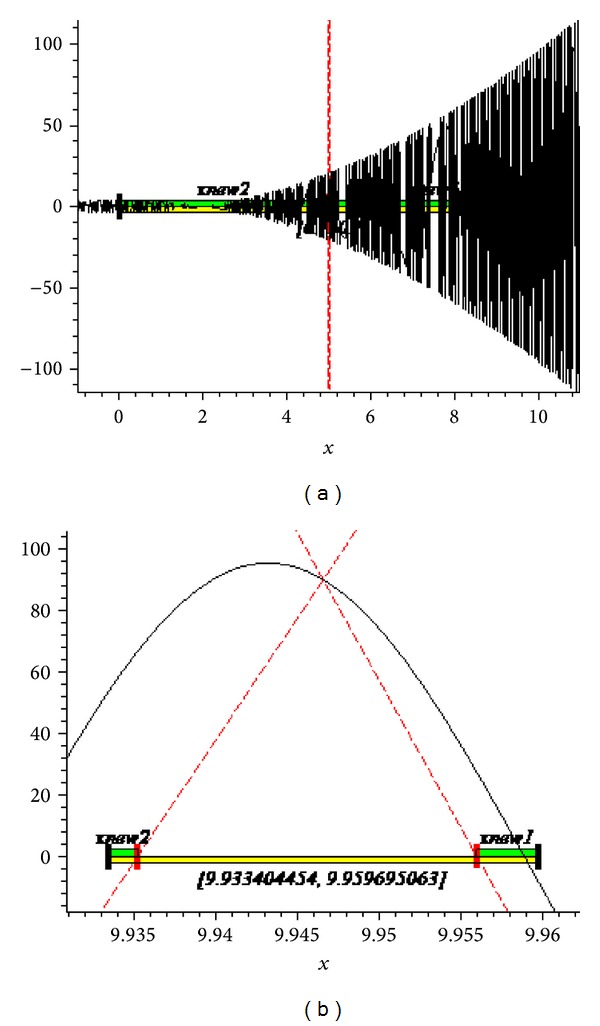
Graph of first and tenth iteration of extended interval Newton's method for *f*_8_(*x*).

**Figure 3:**
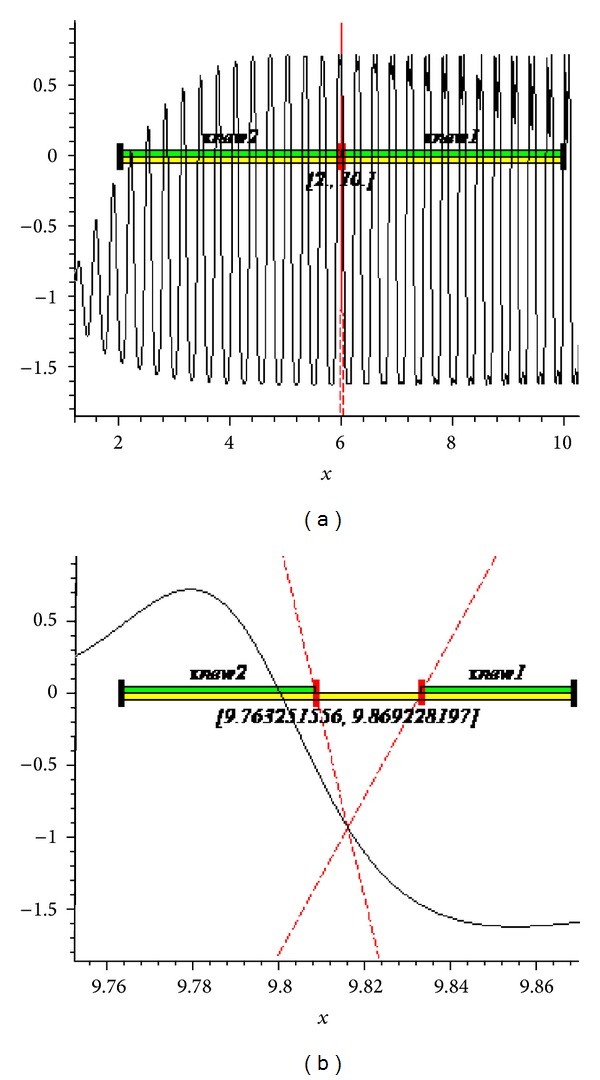
Graph of first and tenth iteration of extended interval Newton's method for *f*_9_(*x*).

The graphs of the first and tenth iteration of extended interval Newton's method for each function obtained by using Maple are shown in Figures 1, 2, and 3, representing the enclosure of exact zeroes at each interval.

## 6. Basins of Attraction

We consider complex polynomials *p*_*n*_(*x*), *n* ≥ 1, *x* ∈ *C*. To generate basins of attraction, we use two different techniques. In the first technique, we take a square box of [−2,2] × [−2,2] ∈ *C*. Now for every initial guess *x*_0_, we assign a colour according to the root to which an iterative method converges. For divergence, we assign the colour dark blue. The stopping criteria for convergence are |*f*(*x*_*k*_)| < 10^−5^, and the maximum number of iterations is 30. In the second technique, we take the same scale, but we assign a colour for each initial guess depending upon the number of iterations in which the iterative method converges to any of the roots of the given function. The maximum number of iterations taken here is 25; stopping criterion is same as given earlier. If an iterative method does not converge in the maximum number of iterations, we consider the method as divergent for that initial guess and the method thus is represented by black colour.

To obtain basins of attraction, we take four test examples which are given as *p*_3_(*x*) = *x* ^3^ − 1, *p*_4_(*x*) = *x* ^4^ − 10*x* ^2^ + 9, *p*_5_(*x*) = *x* ^5^ − 1, and *p*_7_(*x*) = *x* ^7^ − 1. The roots of *p*_3_(*x*) are 1.0, −0.5000 + 0.86605*I*, −0.5000 − 0.86605*I*; roots of *p*_4_(*x*) are −3, 3, −1, 1, and for *p*_5_(*x*) roots are 1.0, 0.3090 + 0.95105*I*, − 0.8090 + 0.58778*I*, −0.8090 − 0.58778*I*, 0.30902 − 0.95105*I*. And roots of *p*_7_(*x*) are 1.0, 0.6234 + 0.78183*I*, −0.2225 + 0.97492*I*, −0.9009 + 0.43388*I*, −0.9009 − 0.43388*I*, −0.2225 − 0.974927*I*, 0.6234 − 0.781831*I*.

We compare the results of our newly constructed method M1 with those of well-known sixteenth-order convergent methods PF [5], JR [1], and FS [2] (see Figures 4, 5, 6, 7, 8, 9, 10, 11, 12, 13, 14, 15, 16, 17, 18, and 19).

## 7. Conclusions

A general four-step four-point iterative method without memory has been given for solving nonlinear equations. This iterative method has been obtained by approximating the first derivative of the function at the fourth step by using quasi-Hermite interpolation. An analytic proof for the order of convergence of this method was given which demonstrates that the method has an optimal order of sixteen. For this method the number of function evaluations is five per full step, so the efficiency index of the method is 1.741. Numerical comparisons in the form of Tables 2, 3, 4, 5, 6, and 7 with the methods based on rational interpolation, that is, methods with comparably more arithmetic cost as shown in Table 9, reveal the robust performance of this method compared to existing methods of this domain. Moreover, extended interval Newton's method is also introduced which is very effective in finding enough accurate initial guesses for solving nonlinear functions having finitely many zeroes in an interval. The basins of attraction show that our new method requires less number of iterations to converge to a root compared to the methods of [2, 5].

## Figures and Tables

**Figure 4:**
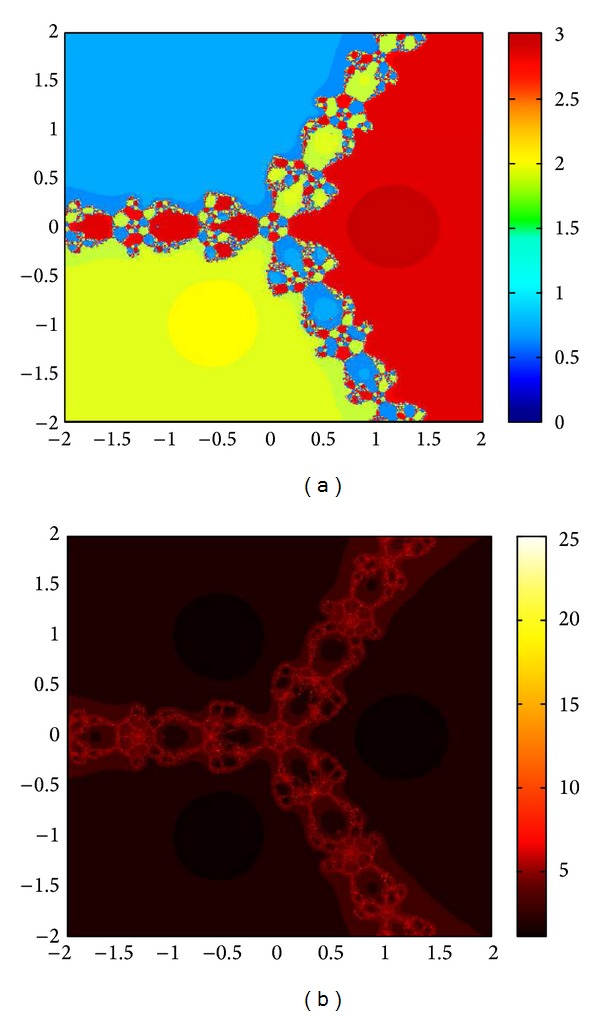
Basins of attraction of method (28) for *p*_3_(*x*).

**Figure 5:**
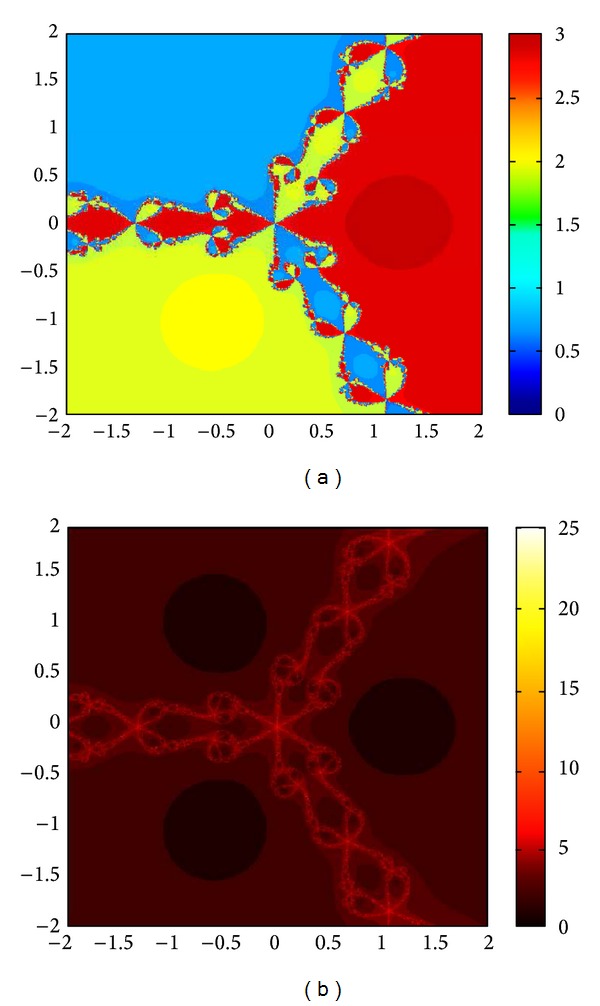
Basins of attraction of method PF for *p*_3_(*x*).

**Figure 6:**
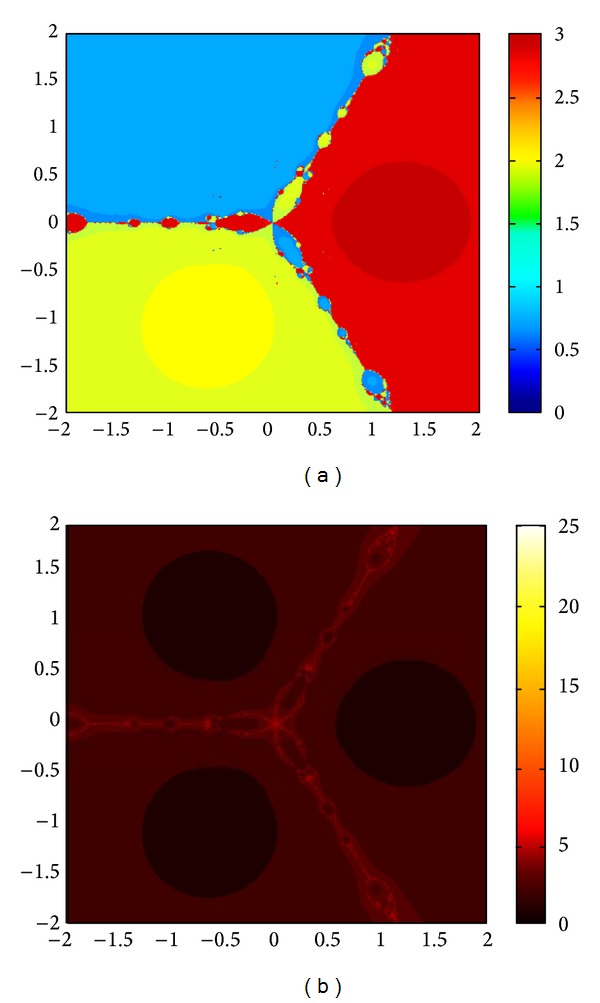
Basins of attraction of method JRP for *p*_3_(*x*).

**Figure 7:**
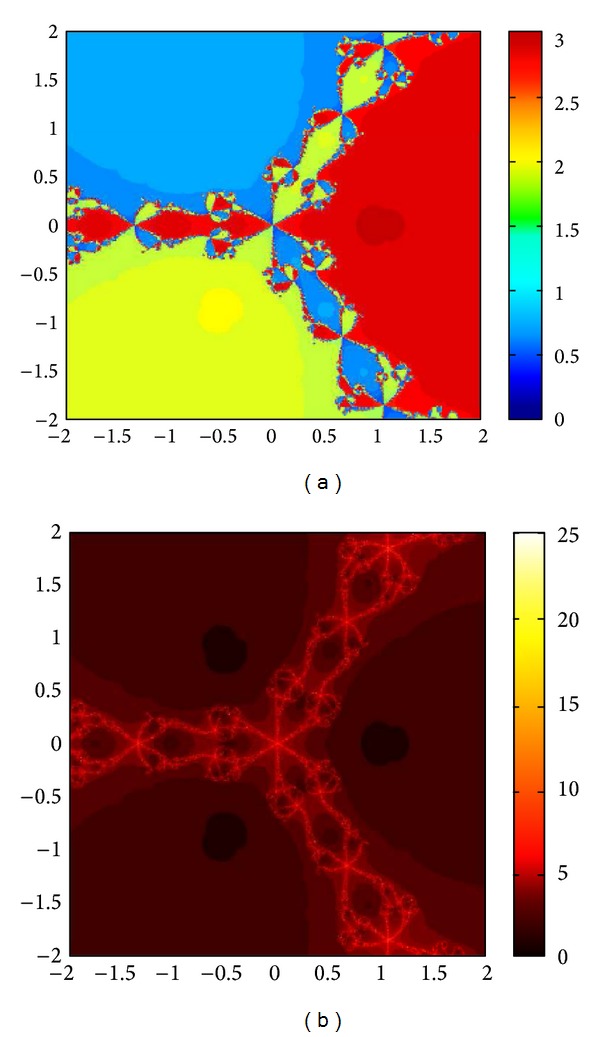
Basins of attraction of method FSH for *p*_3_(*x*).

**Figure 8:**
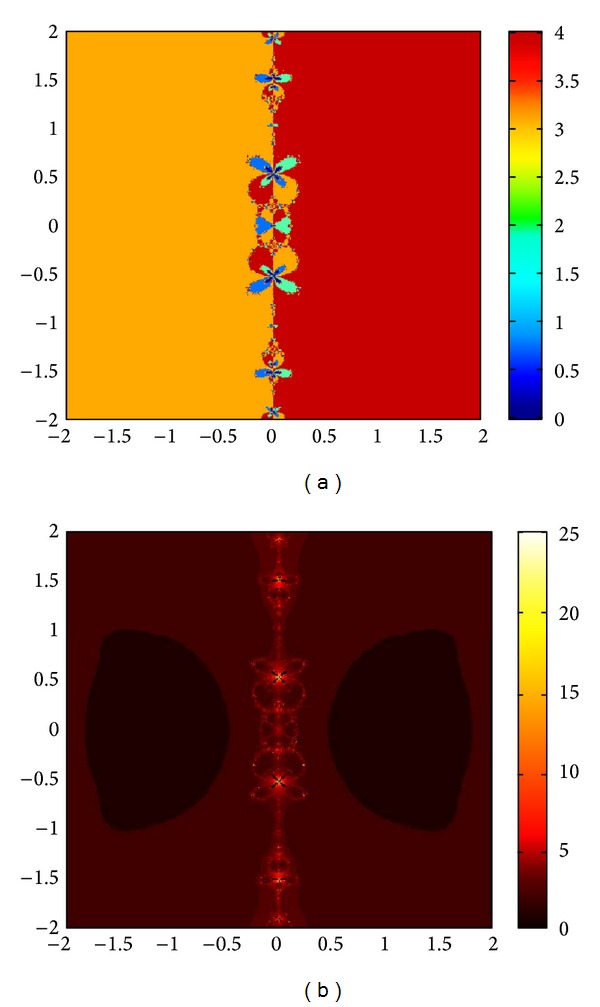
Basins of attraction of method (28) for *p*_4_(*x*).

**Figure 9:**
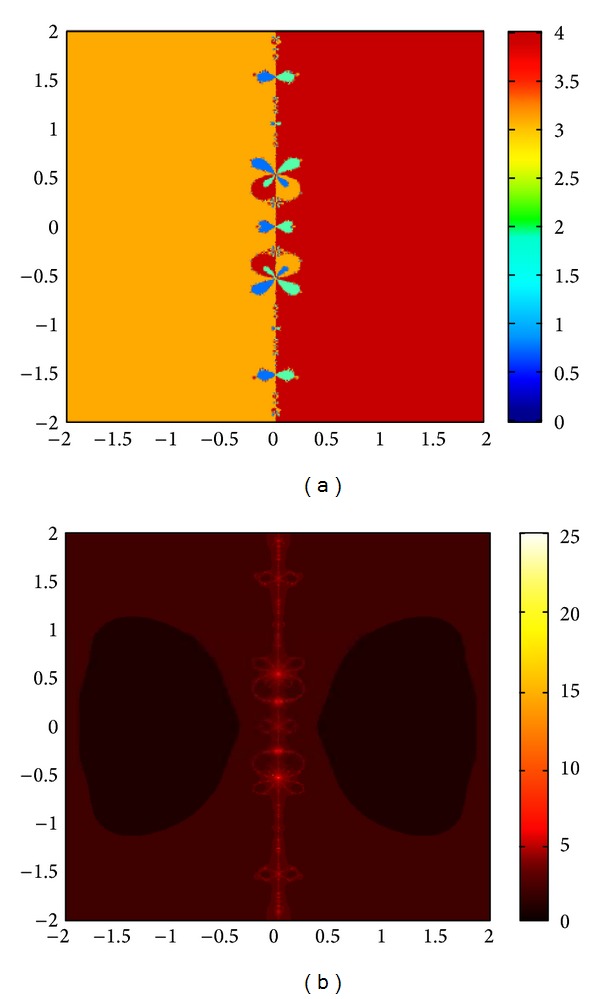
Basins of attraction of method PF for *p*_4_(*x*).

**Figure 10:**
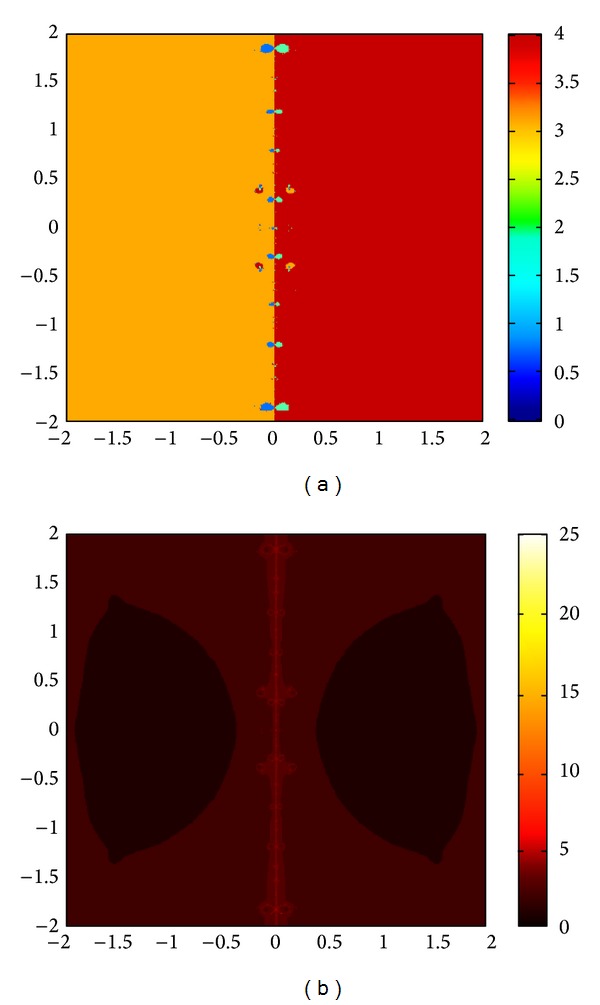
Basins of attraction of method JRP for *p*_4_(*x*).

**Figure 11:**
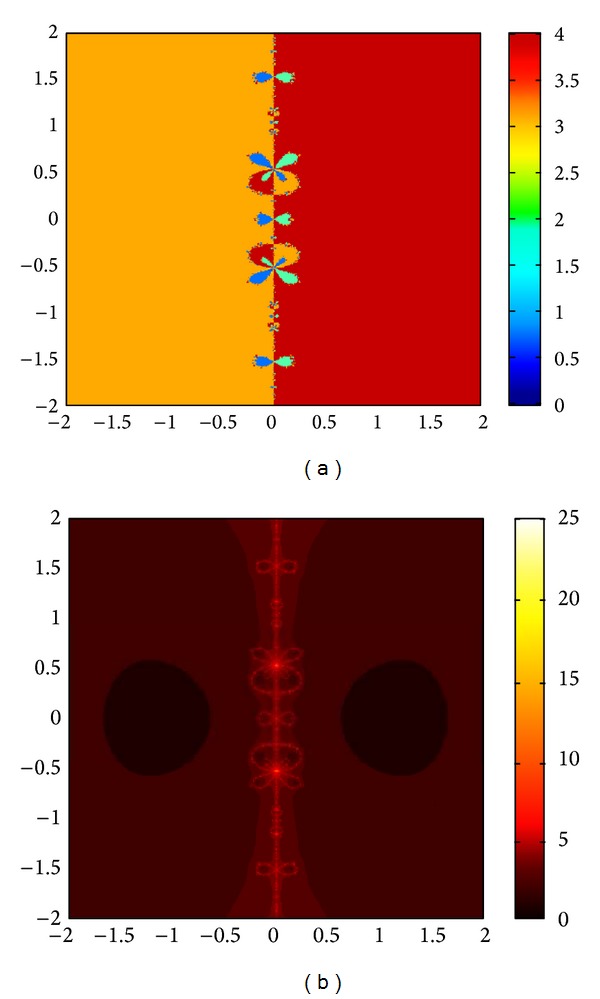
Basins of attraction of method FSH for *p*_4_(*x*).

**Figure 12:**
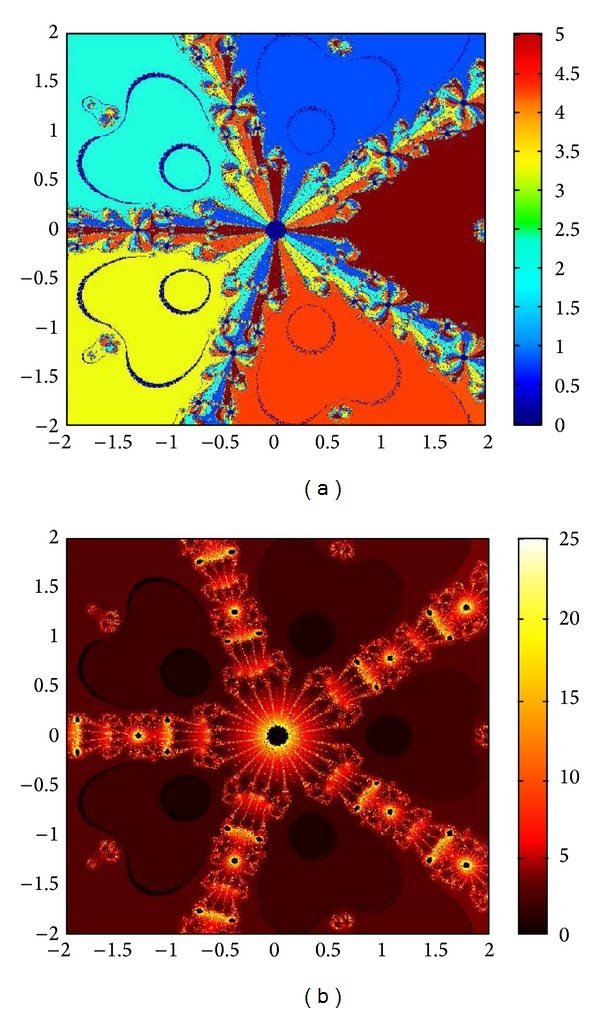
Basins of attraction of method (28) for *p*_5_(*x*).

**Figure 13:**
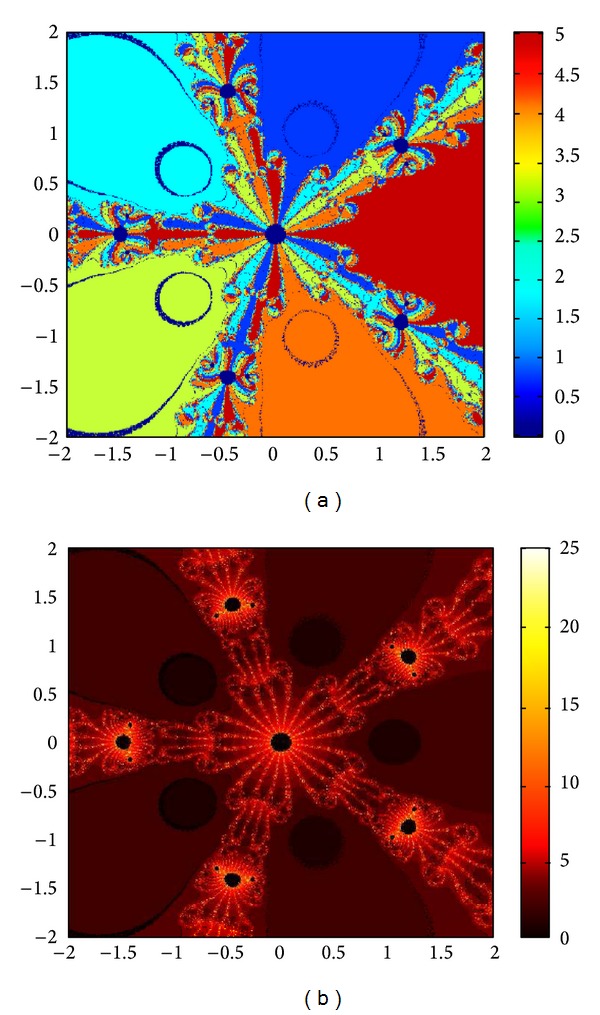
Basins of attraction of method PF for *p*_5_(*x*).

**Figure 14:**
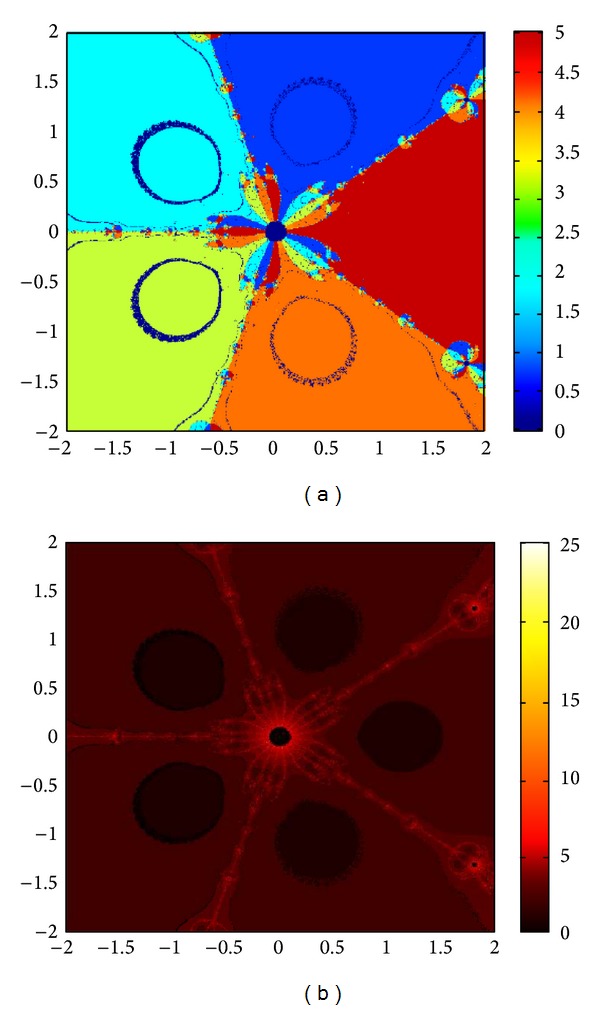
Basins of attraction of method JRP for *p*_5_(*x*).

**Figure 15:**
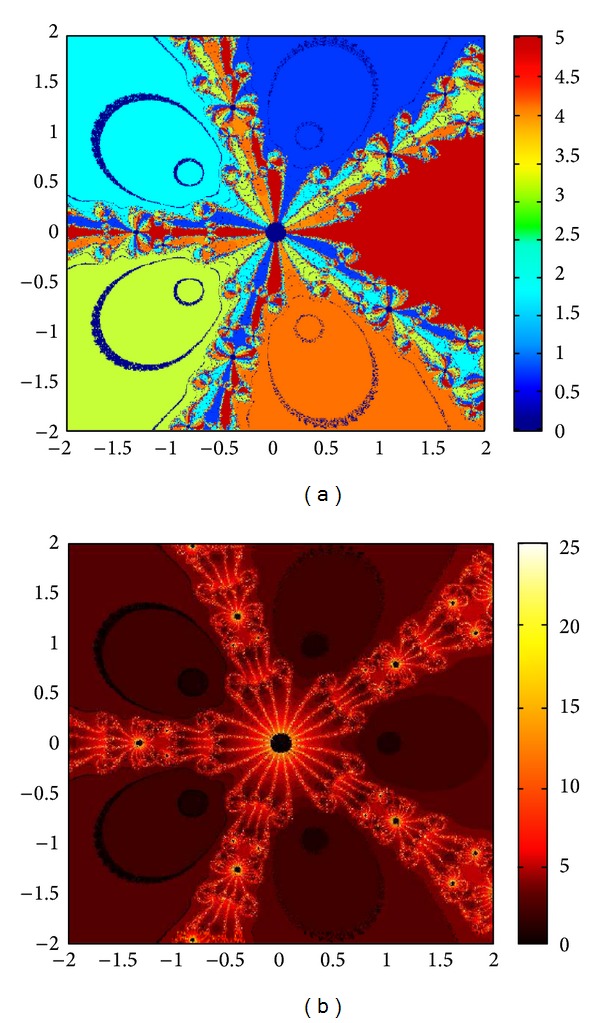
Basins of attraction of method FSH for *p*_5_(*x*).

**Figure 16:**
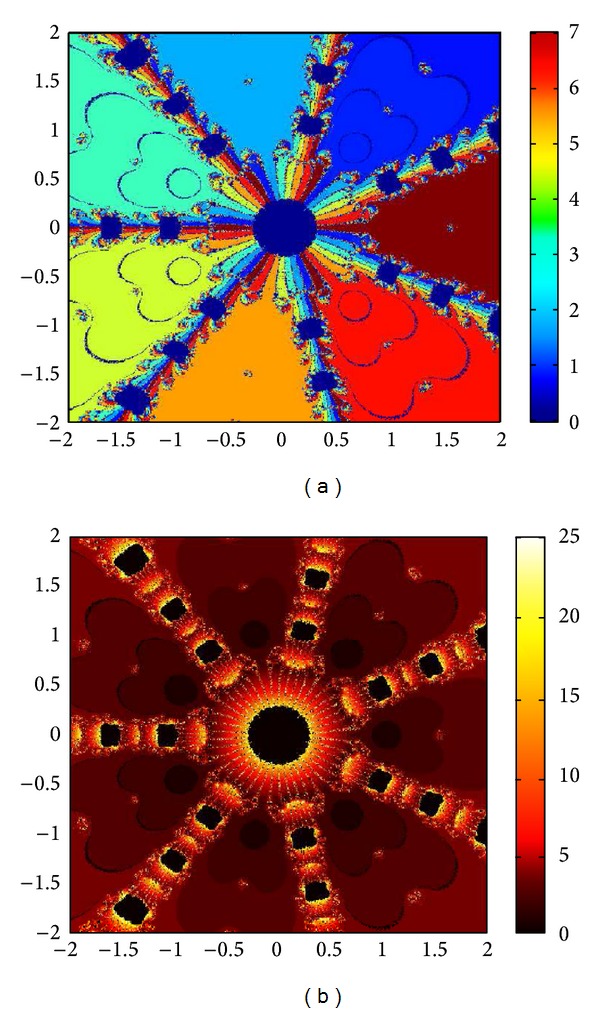
Basins of attraction of method (28) for *p*_7_(*x*).

**Figure 17:**
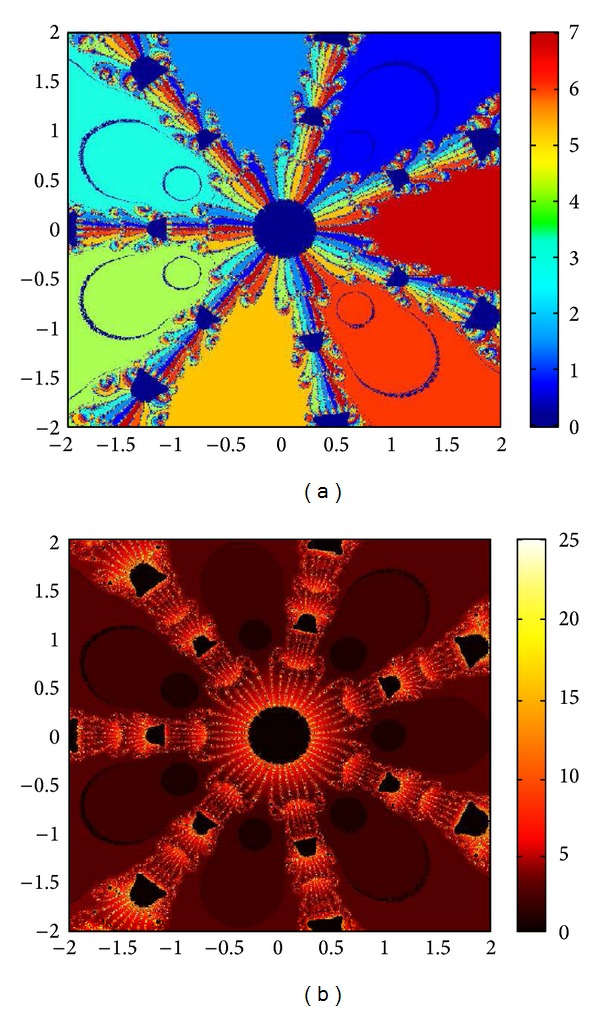
Basins of attraction of method PF for *p*_7_(*x*).

**Figure 18:**
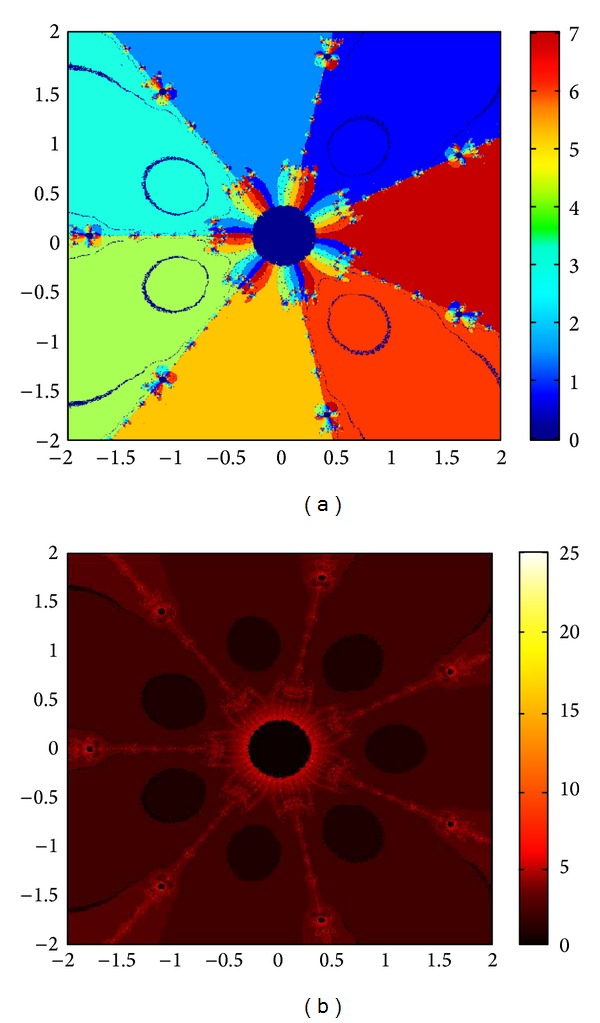
Basins of attraction of method JRP for *p*_7_(*x*).

**Figure 19:**
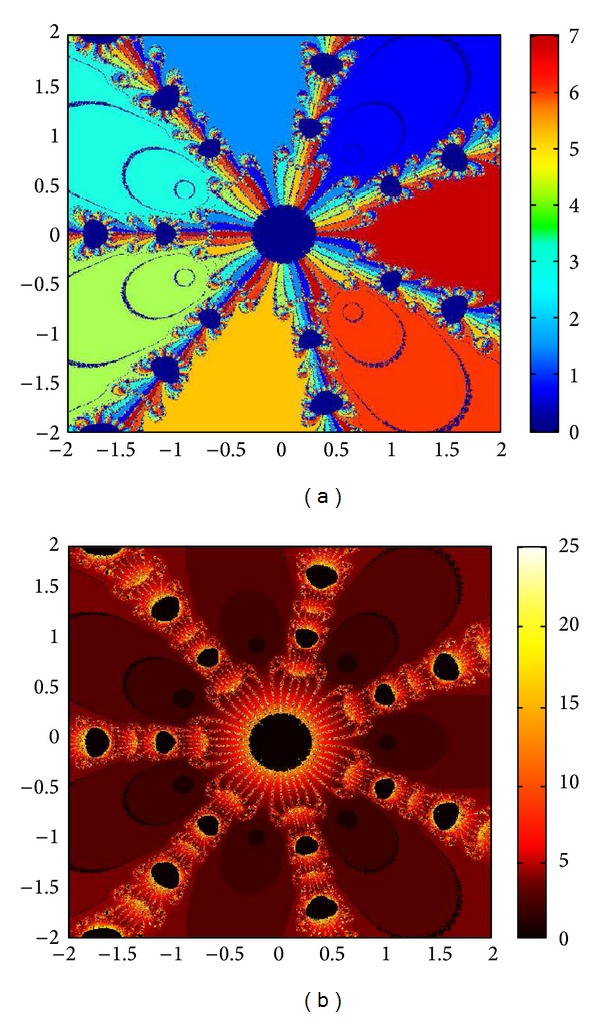
Basins of attraction of method FSH for *p*_7_(*x*).

**Table 1:** Numerical examples.

Numerical example	Exact zero
$\;f_{1}\left(x\right)=\sin\left(x\right)-\frac{x}{100}$	x* = 0.0000000000000000
$f_{2}\left(x\right)=e^{\sin\left(x\right)}-1-\frac{x}{5}$	x* = 0.0000000000000000
*f*_3_(*x*) = *e* ^−*x*^ + cos⁡(*x*)	x* = 1.7461395304080130
*f*_4_(*x*) = *x* ^3^ + 4*x* ^2^ − 15	x* = 1.6319808055660635
*f*_5_(*x*) = 10*xe* ^−*x*^2^^ − 1	x* = 1.6796306104284499
$f_{6}\left(x\right)=\;\sqrt{x^{2}+2x+5}-2\sin\left(x\right)-x^{2}+3\;$	x* = 2.3319676558839640

**Table 2:** Comparison table for *f*_1_(*x*).

f_1_(x), x_0_ = −0.9	n	|f_1_(x_1_)|	|f_1_(x_2_)|	|f_1_(x_3_)|
PF	3	0.602 10^−5^	0.244 10^−105^	0.907 10^−2013^
JRP	3	0.234 10^−5^	0.205 10^−124^	0.133 10^−2624^
FSH	3	0.149 10^−5^	0.286 10^−129^	0.248 10^−2727^
M1	3	0.138 10^−5^	0.561 10^−130^	0.349 10^−2742^
M2	3	0.419 10^−7^	0.737 10^−162^	0.107 10^−3411^

**Table 3:** Comparison table for *f*_2_(*x*).

f_2_(x), x_0_ = 1.0	n	|f_2_(x_1_)|	|f_2_(x_2_)|	|f_2_(x_3_)|
PF	3	0.178 10^−6^	0.455 10^−94^	0.227 10^−1320^
JRP	3	0.815 10^−9^	0.143 10^−146^	0.124 10^−2350^
FSH	3	0.375 10^−8^	0.299 10^−126^	0.139 10^−1899^
M1	3	0.239 10^−8^	0.610 10^−137^	0.192 10^−2194^
M2	3	0.202 10^−8^	0.631 10^−141^	0.510 10^−2261^

**Table 4:** Comparison table for *f*_3_(*x*).

f_3_(x), x_0_ = 0.5	n	|f_3_(x_1_)|	|f_3_(x_2_)|	|f_3_(x_3_)|
PF	3	0.628 10^−7^	0.899 10^−116^	0.197 10^−1748^
JRP	3	0.992 10^−9^	0.111 10^−152^	0.734 10^−2456^
FSH	3	0.335 10^−8^	0.701 10^−145^	0.942 10^−2332^
M1	3	0.143 10^−8^	0.850 10^−151^	0.204 10^−2426^
M2	3	0.103 10^−8^	0.197 10^−153^	0.655 10^−2469^

**Table 5:** Comparison table for *f*_4_(*x*).

f_4_(x), x_0_ = 3.0	n	|f_4_(x_1_)|	|f_4_(x_2_)|	|f_4_(x_3_)|
PF	3	0.00023181	0.471 10^−81^	0.410 10^−1324^
JRP	3	0.00001307	0.455 10^−103^	0.214 10^−1678^
FSH	3	0.00023181	0.471 10^−81^	0.410 10^−1324^
M1	3	0.00023181	0.471 10^−81^	0.410 10^−1324^
M2	3	0.890 10^−5^	0.159 10^−106^	0.180 10^−1734^

**Table 6:** Comparison table for *f*_5_(*x*).

f_5_(x), x_0_ = 0.0	n	|f_5_(x_1_)|	|f_5_(x_2_)|	|f_5_(x_3_)|
PF	3	0.322 10^−17^	0.430 10^−260^	0.247 10^−3660^
JRP	3	0.186 10^−19^	0.128 10^−332^	0
FSH	3	0.101 10^−19^	0.230 10^−337^	0
M1	3	0.955 10^−20^	0.884 10^−338^	0
M2	3	0.860 10^−20^	0.511 10^−339^	0

**Table 7:** Comparison table for *f*_6_(*x*).

f_6_(x), x_0_ = 2.0	n	|f_6_(x_1_)|	|f_6_(x_2_)|	|f_6_(x_3_)|
PF	3	0.125 10^−15^	0.336 10^−236^	0.354 10^−3324^
JRP	3	0.263 10^−18^	0.273 10^−313^	0
FSH	3	0.346 10^−18^	0.549 10^−311^	0.2 10^−3998^
M1	3	0.138 10^−18^	0.119 10^−317^	0
M2	3	0.156 10^−18^	0.165 10^−316^	0

**Table 8:** Comparison table for *f*_7_(*x*),  *f*_8_(*x*), and *f*_9_(*x*).

f(x)		NM	M1	M2
*f*_7_(*x*)	*n*	10	3	3
*x*_0_ = 3.2	|*f*_7_(*x*_*n*_)|	0.118 10^−1013^	0.135 10^−3415^	0.243 10^−3666^
	x*	3.253180973	3.253180973	3.253180973

*f*_7_(*x*)	*n*	13	4	4
*x*_0_ = 3.0	|*f*_7_(*x*_*n*_)|	0.126 10^−1768^	0.10 10^−3998^	0.10 10^−3998^
	x*	3.253180973	3.253180973	3.253180973

*f*_7_(*x*)	*n*	—	—	—
*x*_0_ = 0.9	|*f*_7_(*x*_*n*_)|	—	—	—
	x*	D	D	D

*f*_8_(*x*)	*n*	10	3	3
*x*_0_ = 2.8	|*f*_8_(*x*_*n*_)|	0.126 10^−1765^	0.115 10^−3996^	0.115 10^−3996^
	x*	2.796017462	2.796017462	2.796017462

*f*_8_(*x*)	*n*	11	4	3
*x*_0_ = 2.4	|*f*_8_(*x*_*n*_)|	0.221 10^−1921^	0.726 10^−3997^	0.706 10^−1061^
	x*	2.481858196	2.481858196	2.387610416

*f*_8_(*x*)	*n*	12	4	3
*x*_0_ = 14.0	|*f*_8_(*x*_*n*_)|	0.587 10^−1226^	0.594 10^−3994^	0.580 10^−1410^
	x*	14.01150324	14.01150324	14.01150324

*f*_9_(*x*)	*n*	11	3	3
*x*_0_ = 2.18	|*f*_9_(*x*_*n*_)|	0.170 10^−1209^	0.175 10^−2003^	0.156 10^−2402^
	x*	2.188557091	2.188557091	2.188557091

*f*_9_(*x*)	*n*	12	4	4
*x*_0_ = 2.15	|*f*_9_(*x*_*n*_)|	0.120 10^−1044^	0.10 10^−3998^	0.10 10^−3998^
	x*	2.188557091	2.188557091	2.188557091

*f*_9_(*x*)	*n*	—	—	—
*x*_0_ = −1.5	|*f*_9_(*x*_*n*_)|	—	—	—
	x*	D	D	D

∗D stands for divergence.

**Table 9:** Comparison of computational costs.

	Order	Addition/subtraction	Multiplication/division	Total
PF	14	27	21	48
JR	16	30	41	71
FS	16	69	66	135
M1	16	32	35	67
M2	16	31	34	65

## References

[1] Sharma JR, Guha RK, Gupta P (2013). Improved King's methods with optimal order of convergence based on rational approximations. *Applied Mathematics Letters*.

[2] Soleymani F, Shateyi S, Salmani H (2012). Computing simple roots by an optimal sixteenth-order class. *Journal of Applied Mathematics*.

[3] Petković MS (2010). On a general class of multipoint root-finding methods of high computational efficiency. *SIAM Journal on Numerical Analysis*.

[4] Petković MS (2011). Remarks on ‘On a general class of multipoint root-finding methods of high computational efficiency’. *SIAM Journal on Numerical Analysis*.

[5] Sargolzaei P, Soleymani F (2011). Accurate fourteenth-order methods for solving nonlinear equations. *Numerical Algorithms*.

[6] Traub JF (1964). *Iterative Methods for the Solution of Equations*.

[7] Geum YH, Kim YI (2010). A multi-parameter family of three-step eighth-order iterative methods locating a simple root. *Applied Mathematics and Computation*.

[8] King RF (1973). A family of fourth order methods for nonlinear equations. *SIAM Journal on Numerical Analysis*.

[9] Soleymani F, Babajee DKR, Shateyi S, Motsa SS (2012). Construction of optimal derivative-free techniques without memory. *Journal of Applied Mathematics*.

[10] Moore RE, Kearfott RB, Cloud MJ (2009). *Introduction to Interval Analysis*.

